# Apothesine Anesthesia

**Published:** 1918-12

**Authors:** O. O. Carter

**Affiliations:** Indianapolis, Ind.


### APOTHESINE ANESTHESIA

BY O. O. CARTER, D.D.S., INDIANAPOLIS, IND.

In selecting a local anesthetic for dental operative work I have always considered three factors to be of paramount importance.

First, its anesthetic properties: If an agent will not produce complete desensitization within a reasonable period of time the results are nothing short of a failure. Unless the operator can proceed with full confidence that his patient will suffer no appreciable amount of pain during the operation he will be unable to work in a manner satisfactory to himself and to the patient.

Second, toxicity: This is prompted by the alarming symptoms or disastrous effects of cocaine solution in certain instances, that have been recorded in medical and dental literature. No doubt there have been many more cases that have never become a matter of record.

Third, sloughing, not always the least in importance: It is a well known fact that the tissues into which a dentist elects to inject any anesthetic solution are very susceptible to pressure or to chemical irritation. While it is true that sloughing and necrosis may sometimes follow the injection of small quantities of sterile water ( normal salt solution, such conditions are extremely rare and usually anticipated after carefully examining the tissues before making the injection. This being true, it is desirable to select for anesthetic purposes an agent that offers the least possible danger of affecting the tissues in any way that might produce sloughing.

During the past year I have been using apothesine, almost to the exclusion of other local anesthetics. Apothesine is a synthetic drug in the form of white crystals which are readily soluble in water. It is claimed to be less toxic than novocaine. It is stable under ordinary conditions, and may be sterilized safely by heating to the boiling point. It is marketed by Parke, Davis & Co., in one and one-fourth grain tablets, which can be dissolved in normal salt solution to a two per cent. saturation for injection. Another form of tablets may be had which contains three-fifths of a grain of apothesine and one sixteen-hundredths of a grain of adrenaline. One of these tablets dissolved in fifty drops of water or normal salt solution will make a one per cent. solution. The results seem to warrant the conclusion that it fully meets all the requirements for dental operative work. I have used it extensively in all kinds of extracting, removing as many as twenty-one teeth during one operative procedure. In this particular case I injected the solution on both labial and lingual sides of the teeth, making approximately twenty-five injections of a two per cent. apothesine solution, from one to two c. c. for each injection. Following this operation there was no nausea, no noticeable effect on the heart, and the gums healed quickly without sloughing and patient made a prompt recovery.

There has been no temporary or permanent edema observed at point of injection following operations or during convalescence. In removing necrosed bone and treating abscessed cases with apothesine I have had less complications than at any time during my experience with local anesthetics.

For adults I use two per cent. solution and in children one to two per cent. solution, always boiling the solution before using. I have had an extensive experience in extracting for children and in these cases there has never been a complaint of pain nor any harmful effect observed.

Poor technic is often responsible for poor results in the use of any anesthetic solution. If the best results are always to be obtained it is necessary for the operator to employ a sterile solution, sterile syringe and needle, using a small needle with a very sharp point. Make the injections slowly, causing as little traumatism as possible.

